# Synthetic glycolipid-based TLR4 antagonists negatively regulate TRIF-dependent TLR4 signalling in human macrophages

**DOI:** 10.1177/17534259211005840

**Published:** 2021-04-16

**Authors:** Charys Palmer, Fabio A Facchini, Richard PO Jones, Frank Neumann, Francesco Peri, Grisha Pirianov

**Affiliations:** 1 School of Life Sciences, Anglia Ruskin University, UK; 2 Department of Biotechnology and Biosciences, University of Milano-Bicocca, Italy; 3 Innaxon Biosciences, UK

**Keywords:** Toll-like receptor 4, TLR4, TRIF, drug development, inflammation, THP-1 macrophages, IFN-β, IP-10

## Abstract

TLRs, including TLR4, play a crucial role in inflammatory-based diseases, and TLR4 has been identified as a therapeutic target for pharmacological intervention. In previous studies, we investigated the potential of FP7, a novel synthetic glycolipid active as a TLR4 antagonist, to inhibit haematopoietic and non-haematopoietic MyD88-dependent TLR4 pro-inflammatory signalling. The main aim of this study was to investigate the action of FP7 and its derivative FP12 on MyD88-independent TLR4 signalling in THP-1 derived macrophages. Western blotting, Ab array and ELISA approaches were used to explore the effect of FP7 and FP12 on TRIF-dependent TLR4 functional activity in response to LPS and other endogenous TLR4 ligands in THP-1 macrophages. A different kinetic in the inhibition of endotoxin-driven TBK1, IRF3 and STAT1 phosphorylation was observed using different LPS chemotypes. Following activation of TLR4 by LPS, data revealed that FP7 and FP12 inhibited TBK1, IRF3 and STAT1 phosphorylation which was associated with down-regulation IFN-β and IP-10. Specific blockage of the IFN type one receptor showed that these novel molecules inhibited TRIF-dependent TLR4 signalling via IFN-β pathways. These results add novel information on the mechanism of action of monosaccharide FP derivatives. The inhibition of the TRIF-dependent pathway in human macrophages suggests potential therapeutic uses for these novel TLR4 antagonists in pharmacological interventions on inflammatory diseases.

## Introduction

The incidence of inflammatory-based diseases has increased dramatically due to a variety of environmental factors. Over the last few decades, a solid body of research papers have identified many therapeutic targets and anti-inflammatory compounds which have been validated for treatment of these disorders. Lack of target specificity and side effects are the current problems hampering clinical application of small molecular anti-inflammatory drugs.^1–3^ Discovery of novel target-specific compounds for treatment of these diseases is a big challenge with potentially significant scientific, commercial and social impacts. TLRs are PRRs within the immune system, and TLR recognition of microbial PAMP or endogenous DAMP (danger-associated molecular patterns) leads to the activation of inflammation and triggering of the innate immune response.^4–5^ TLR4 is the main sensor specific for LPS that represents the main outer membrane component of most Gram-negative bacteria.^6^ TLR4 activation by endogenous ligands (DAMPs) and signalling has been implicated in the pathogenesis of a number of inflammatory-related diseases.^7^ Following ligand recognition, TLR4 may activate MyD88-dependent or TRIF-dependent signalling pathways. MyD88-dependent signalling is associated with downstream activation of NF-kB and production of pro-inflammatory cytokines and chemokines.^9^ TRIF-dependent signalling is typically associated with IRF3 and production of type 1 IFN, as well as late phase NF-kB activation via TRAF6.^9^ While TLR3/TRIF-dependent signalling may have some protective effect against cardiovascular disease, TLR4/TRIF-dependent signalling is considered pro-atherogenic.^10^ Therefore, TLR4-specific antagonists of TRIF signalling are also of great interest for treatment of these diseases. The deletion of the TLR4 gene in haematopoietic and non-haematopoietic cells protected against a variety of inflammatory-based diseases.^11–13^ These findings strongly support the idea that regulation of TLR4 may be a promising target for therapeutic control of inflammatory-related disorders.^14^

Over the last two decades, TLR4 antagonists have been evaluated in pre-clinical and clinical studies; however, none have been approved yet for clinical use. Therefore, the discovery of novel TLR4 modulators is an important target for the pharmaceutical industry. Recently, we have developed synthetic glycolipids, named FP7 and FP12, as TLR4 antagonists.^15^ FP7 and FP12 compete with LPS (and other ligands) for the MD-2 binding site, thus inhibiting TLR4 activation (formation of the TLR4/MD-2/LPS complex).^15^ In a previous study we have shown the ability of FP7 to negatively regulate MyD88-dependent TLR4 signalling in both non-haematopoietic and haematopoietic cells, which suggests that this TLR4 antagonist could potentially be used therapeutically for the treatment of inflammatory-related diseases.^16^ The main aim of this study was to further investigate the potential of these novel molecules to affect MyD88-independent or TRIF-dependent TLR4 signalling pathways in THP-1 macrophages. Our results identified specific targets for FP7 and FP12, by which they inhibited LPS-driven TRIF-dependent TLR4 signalling in human macrophages.

## Materials and methods

### Materials

TLR4 antagonists FP7 and FP12 were synthesized in Prof F Peri laboratories (University of Milano-Bicocca) by multistep organic synthesis and the purity and identity of the compounds was confirmed by NMR, mass spectrometry and HPLC analyses.^16^ For TLR4-exclusive and potent activation, LPS (*Salmonella* Minnesota (SM) (Re), TLRpure™), LPS (SM (Ra), TLRpure™), LPS (SM (S-form), TLRpure™), Lipid A (SM, TLRpure™) and LPS from *Escherichia coli* (Re) were used (Innaxon Biosciences, Tewkesbury, UK). For *in vitro* experiments FP7 and FP12 were reconstituted in DMSO/ethanol (1:1) (vol: vol). Anti-human IFNR2 neutralizing Ab (clone MMHAR-20) was purchased from PBL Assay Science, USA.

### Cell maintenance and treatment

THP-1 cells were obtained from the European Collection of Animal Cell Cultures (Salisbury, Wiltshire, UK) and cultured in RPMI (+10% heat inactivated FBS, (HIFBS)), +1% glutamine, +1% penicillin/streptomycin). Cells were split three times weekly and maintained at a density of ∼0.3 × 10^6^ cells/ml. For differentiation of THP-1 cells, 25 nM of PMA (phorbol 12-myristate 13-acetate) was added to plated cells for 3 d before washing three times with fresh medium. Cells were then left to rest overnight before treatment. All cells were pre-treated with FP7 and FP12 (10 μM) for 1 h, then exposed to LPS (100 ng/ml) for 0 to 16 h. In some experiments, cells were simultaneously incubated with FP (Francesco Peri) compounds and LPS, or FP compounds were added to culture medium 30 min after LPS.

### Western blot analysis of protein expression and phosphorylation

Cell lysates (50 μg) were separated on 7.5% TGX gels, transferred onto polyvinyldifluoride membranes (Bio-Rad, UK) and blocked using 5% (wt/vol) skimmed milk in tris-buffered saline (TBS)/0.1% (vol/vol) tween-20 for 1 h at room temperature. Blots were incubated overnight at 4°C with primary Abs: phospho-p38 (4511), phospho-p65 NF-kB (3031), β-actin (12262), phospho-TBK1 (5483), phospho-IRF3 (4947) or phospho-STAT1 (9167) from Cell Signalling Technology (NEB, Herts, UK) (1:1000 dilution in TBS, 1% milk). After washing in TBS/0.1% (vol/vol) tween-20, blots were incubated with HRP-conjugated secondary Ab at room temperature for 1 h in TBS/0.1% (vol/vol) tween-20 and 5% milk. After the final wash, immunoreactivity was visualized using the chemiluminescent substrate ECL Plus (Bio-Rad, UK). G-Box imaging system and Genesys software (Synoptics UK) were used to visualize blots and densitometry analysis was performed using Genetools (Synoptics UK). The level of cellular beta actin was used as a loading control.

### Inflammation Ab array

THP-1 macrophages were treated with FP7 or FP12 (10 µM) for 1 h prior to LPS (100 ng/ml) exposure. Culture medium was collected after 18 h of incubation and analysed on a human inflammation array (Ray-Biotech, USA) containing 40 pro-inflammatory proteins to assess relative levels of cytokine expression by samples.^16^ Results values are expressed as fold-increase relative to control samples.

### ELISA

Human IFN-β and IP-10 production were measured in cell culture medium (10–100 μl) using ELISA kits (R&D Systems, USA and Ray-Biotech, USA) following the manufacturer’s instructions. At the final stage, absorbance was measured at 450 nm using the Sunrise (Tecan Group LTD., Switzerland) microplate reader. Protein concentration was calculated using GraphPad Prism version 7.01.

### Statistical analysis

Results were analysed by one-way ANOVA followed by the post-hoc test (Tukey) for multiple comparisons using GraphPad Prism version 7.01. A value of *P* < 0.05 was considered significant.

## Results

### Time-dependent activation of TBK1 and STAT1 in TRIF-dependent TLR4 signalling in response to a selection of TLR4-selective agonists in THP-1 macrophages

TLR4 signalling has been shown to play a critical role in the functional activity of immune-competent cells at any stage of the inflammatory process. It has been shown that a prolonged activation of the receptor as a result of stimulation by PAMPs and DAMPs can lead to chronic microbial or sterile inflammations associated with the development and progression of inflammatory diseases. To study the effect of FP7 and FP12 on MyD88-independent TLR4 signalling, THP-1-derived human macrophages were used as an *in vitro* cell model. The experimental design was based on two readouts: the activation of TLR4 second messengers and the production of TLR4-dependent pro-inflammatory proteins associated with this pathway.

To identify a suitable inducer of TRIF-dependent TLR4 signalling, initially we tested the ability of known TLR4 agonists to induce TBK1 and STAT1 phosphorylation.

**Figure 1.**
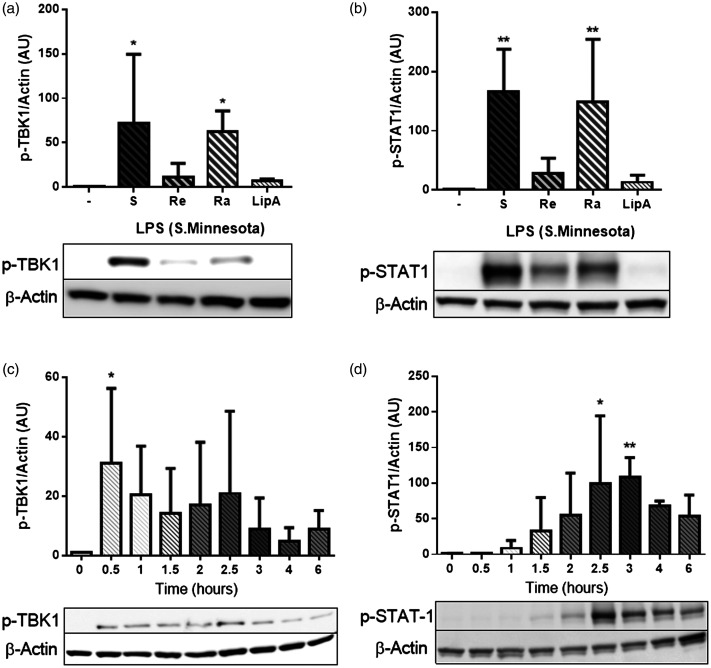
Effect of different TLR4 ligands on TBK1 and STAT1 phosphorylation. (a-b) THP-1-derived macrophages we exposed to LPS (S, Re, Ra), and lipid A (all from SM) and TBK1 and STAT1 phosphorylation was measured by Western blotting at 0.5 and 2.5 h, respectively. (c-d) THP-1-derived macrophages were exposed to LPS (SM, S-form) for 0.5-6 h and TBK1 and STAT1 phosphorylation was measured via Western blotting. Actin was used as a loading control. Results are shown as mean ± SD of three independent experiments. Significant results are indicated as *P < 0.05 and **P < 0.01 versus control.

Deep rough LPS (Re-LPS) from SM, known to potently activate MyD88-dependent TLR4 signalling, did not activate second messengers involved in TRIF-dependent TLR4 signalling (Supplemental Figure 2). Different types of LPS can excite differential effects on TLR4 signalling pathways, and two other forms of SM LPS (S, smooth/wild type and Ra, rough form), as well as with lipid A, were tested. While S-LPS and Ra-LPS produced a significant increase in the levels of TBK1 (Figure 1a) and STAT1 phosphorylation (Figure 1b) compared with the control, no significant effect was seen in response to Re-LPS or lipid A. S-form LPS (SM) was therefore selected for all subsequent experiments on TRIF-dependent TLR4 signalling.

To select the appropriate timing for measurement of phosphorylation events, THP-1 macrophages were exposed to LPS (S-form, SM), and cell lysates were collected over a period of 6 h. Following exposure to LPS (S-form, SM), levels of both TBK1 (Figure 1c) and STAT1 phosphorylation (Figure 1d) were increased. TBK1 phosphorylation was detected at 30 min following LPS treatment, whereas phospho-STAT1 remained below a significant threshold until 2.5–3 h. From this, 30 min and 2.5–3 h were selected for further experiments for measuring TBK1 and STAT1 phosphorylation, respectively. Similarly, we found that phosphorylation of IRF3 (downstream target for TBK1) was elevated between 45–60 min following exposure to LPS (data is not shown). In summary, these results demonstrated substantial differences in the times taken for MyD88 and TRIF-dependent TLR4 signalling.

### FP7 and FP12 suppress LPS-induced TBK1 and IRF3 phosphorylation in THP-1 macrophages

**Figure 2.**
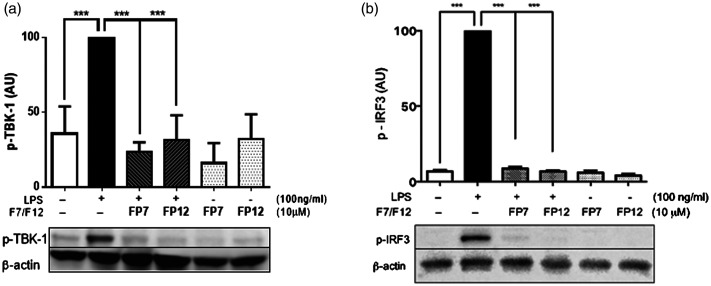
FP7 and FP12 negatively regulate TRIF-dependent TBK1 and IRF3 phosphorylation. THP-1-derived macrophages were pre-treated with FP7 (10 µM) or FP12 (10 µM) for 1 h before exposure to LPS (S-form) (100 ng/ml). Cell lysates were prepared after 0.5 h and TBK1 and IRF3 phosphorylation was measured via Western blotting. Actin was used as a loading control. Results are shown as mean ± SD of three independent experiments. Significant results are indicated as ***P < 0.001 versus control.

We have previously shown that FP7 can reduce MyD88-dependent TLR4 signalling at the second messengers (p65 NF-kB and p38 MAPK) level in THP-1 macrophages.^14^ Similarly to FP7, we found that FP12 can also negatively regulate MyD88-dependent TLR4 signalling at IL-1β level in response to S- and Re-forms of SM LPS (Supplemental Figure 3). However, we wanted to determine the effects of FP7 and FP12 on the alternative TRIF-dependent TLR4 signalling pathway. For this purpose, we analysed their effect on activation of TBK1 and IRF3 as second messengers in this pathway. Following up-regulation of targeted second messengers in response to LPS (S-form, SM), both FP7 and FP12 significantly reduced TBK1 (Figure 2a) and IRF3 (Figure 2b) phosphorylation, to a level comparable with the controls. These results suggested that FP7 and F12 can negatively regulate TRIF-dependent TLR4 signalling at a level of second messengers in THP-1 macrophages.

### Time-dependent production of IFN-β and IP-10 in TLR4 signalling in response to selective TLR4 agonists in THP-1 macrophages

We explored whether FP7 and FP12 could have an impact on LPS-driven production of pro-inflammatory proteins in TRIF-dependent TLR4 signalling in THP-1 macrophages. Initially, we used human inflammation Ab array (containing 40 pro-inflammatory proteins) to screen specific target molecules of interest. The semi-quantitative analysis demonstrated that FP7 and FP12 inhibited, to various extents, the expression of 17/20 LPS(SM)-driven pro-inflammatory proteins including IP-10, which is an IFN-dependent protein the production of which is associated with TLR4/TRIF signalling (Supplemental Table 1). Previously, we have validated the blocking effect of FP7 on the production of TLR4/MyD88-dependent proteins such as IL-1β, IL-6, IL-8, TNFα, MCP-1, MIP-1, etc.^16^ In this study, we investigated the modulating effect of these novel molecules on the production of IFN-related proteins (IFN-β and IP-10). We did not include IFN-γ in the list because LPS did not appear to affect the release of this protein from human macrophages at 24 h.

**Figure 3.**
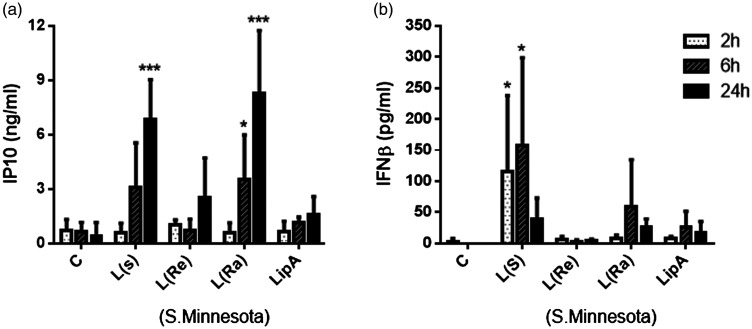
Time-dependent production of IFN-β and IP-10 in TLR4 signalling in response to different chemotypes of LPS as TLR4 selective agonists in THP-1 macrophages. THP-1 macrophages were treated with LPS (100 ng/ml) and lipid A (100 ng/ml) and medium collected at 2, 6 and 24 h. IP-10 (a) and IFN-β (b) release were measured via ELISA. Results are shown as mean ± SD of three independent experiments. Significant results are indicated as *P < 0.05 or ***P < 0.001 versus control.

Furthermore, we applied the same experimental approach as with second messengers. To determine which LPS form was most appropriate for analysing IFN-β and IP-10 production, we used three forms of LPS (S, Re and Ra), as well as lipid A. While S and Ra forms produced a significant increase in production of IP-10 (Figure 3a) and IFN-β (Figure 3b) compared with the control, no significant effect was seen in response to Re-LPS or lipid A.

Hence, the S-form of LPS was selected for all subsequent end point experiments involving TRIF-dependent TLR4 signalling. Additionally, we monitored the time course of IFN-β and IP-10 production. Interestingly, only LPS (S-form) produced a significant increase in IFN-β and IP-10. Following LPS stimulation, we found major differences in the time courses of the release of these IFN proteins. IP-10 was released within 6 h of LPS exposure and the level was further increased at 24 h (Figure 3a). In contrast, the initial increase in IFN-β production at 2–6 h was followed by a drop in IFN-β level by 24 h (Figure 3b). This data demonstrates that a short time of IFN-β availability might be associated with the signalling properties of this molecule through IFN receptor alpha/beta (IFNAR). Following this, experiments involving IP-10 and IFN-β were considered after 24 and 2.5 h of LPS exposure, respectively.

### FP7 and FP12 negatively regulate LPS-induced IFN-β and IP-10 production in THP-1 macrophages

**Figure 4.**
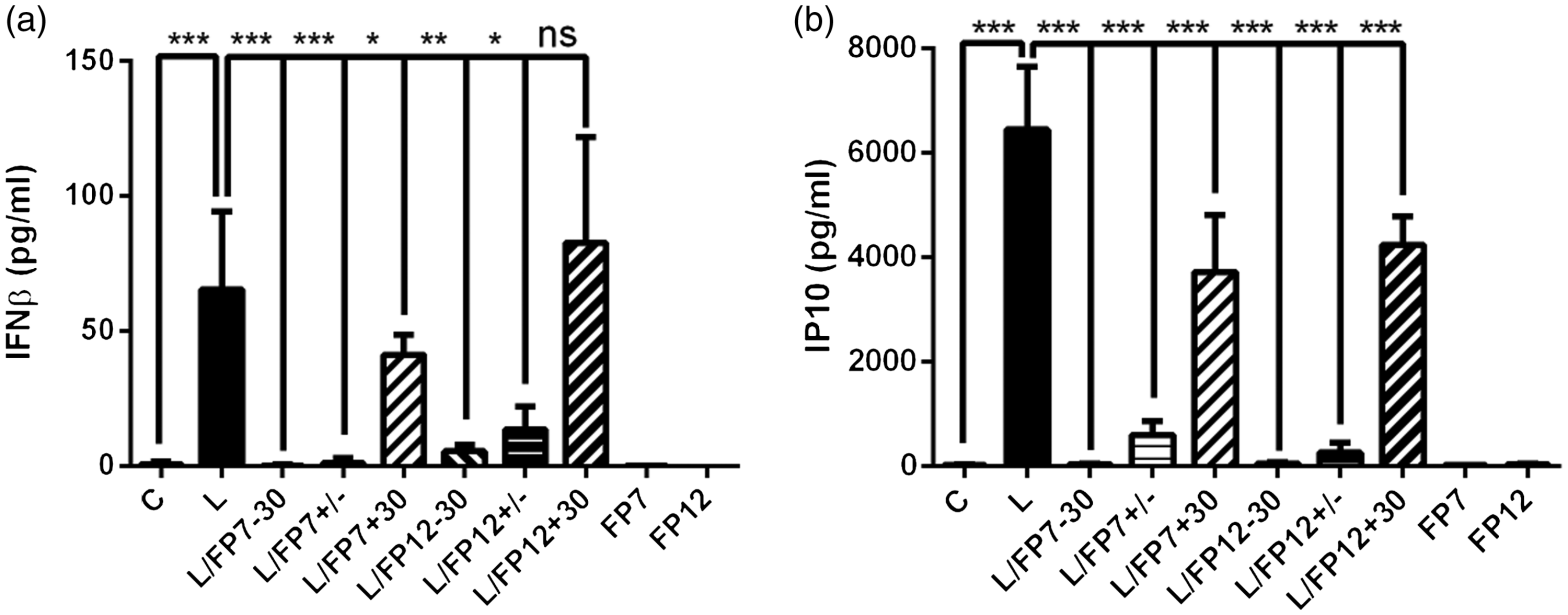
Effect of FP7 and FP12 on IFN-β and IP-10 production in prior, simultaneous and post LPS exposure. THP-1-derived macrophages were pre-treated with FP7 or FP12 30 min before, at the same time, and 30 min after LPS (S-form, 100 ng/ml) exposure. Medium was collected at 2.5 h for IFN-β and 24 h for IP-10. IFN-β (a) and IP-10 (b) release was measured via ELISA. Results are shown as mean ± SD of three independent experiments. Significant results are indicated as *P < 0.05, **P < 0.01 or ***P < 0.001 versus control.

In the next series of experiments, we validated the TLR4-modulating effect of FP7 and FP12 on IFN-β and IP-10 production. THP-1 macrophages were pre-treated with FP7 or FP12 prior to LPS exposure to determine the effect of these molecules on TLR4/TRIF-dependent pro-inflammatory protein production. IFN-β and IP-10 were measured in culture medium after 2.5 and 24 h, respectively. Both FP7 and FP12 significantly down-regulated IFN-β and IP-10 production following LPS (S chemotype) stimulation (Figure 4).

Recently, we have provided evidence demonstrating that, irrespective of the method of administration (prior, simultaneously and even post LPS exposure), FP7 down-regulated MyD88-dependent cytokines production in THP-1 macrophages.^16^ Following the same experimental approach, the results showed the ability of FP7 to reduce TLR4-dependent IFN-β (Figure 4a) or IP-10 (Figure 4b) irrespective of time of the administration of LPS. While FP12 significantly reduced IFN-β production in prior and simultaneous treatments, post LPS treatment with FP12 did not affect IFN-β production (Figure 4a). Similar to FP7, FP12 negatively regulated IP-10 production (Figure 4b). This data suggested that FP7 and FP12 are negative modulators of type I IFN production, however FP12 was found less effective on TRIF-dependent TLR4 signalling *post factum*.

### FP7 and FP12 block LPS-induced STAT1 phosphorylation via IFN-β signalling in THP-1 macrophages

**Figure 5.**
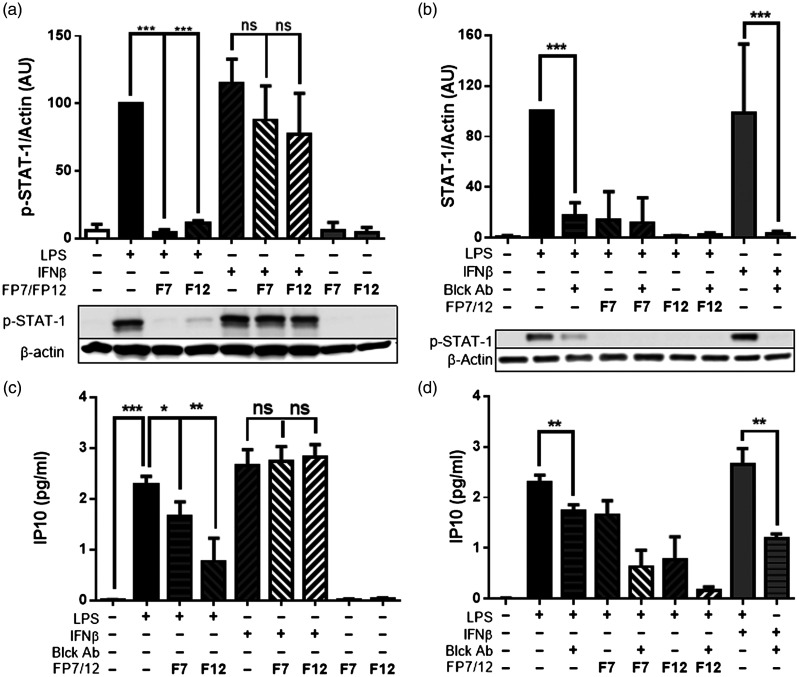
FP7 and FP12 block LPS-induced STAT1 phosphorylation via IFN-β signalling in THP-1-derived macrophages. THP-1 macrophages were pre-treated with FP7, FP12 (10 μM) and IFNAR neutralizing Ab (1 μg/ml) 1 h prior to LPS (S-form) exposure. Cell lysates and medium were collected after 2.5 and 24 h, respectively. STAT1 phosphorylation (a, b) and IP-10 production (c, d) were measured via Western blotting and ELISA, respectively. Results are shown as mean ± SD of three independent experiments. Significant results are indicated as *P < 0.05, **P < 0.01 or ***P < 0.001 versus control.

To further investigate the mechanism by which novel TLR4 antagonists block TLR4/TRIF signalling in macrophages we carried out mechanistic experiments using a specific blocking Ab to prevent IFNAR activation in response to IFN-β. As a readout, we used phosphorylation of STAT1, a well-known second messenger in IFN signalling. Following LPS exposure or direct exogenous IFN-β stimulation, a significant increase in STAT1 phosphorylation was observed (Figure 5a and 5b).

LPS or IFN-β-induced STAT1 phosphorylation was countered by blocking IFNAR in either case, suggesting that IFNAR activation was required for LPS/TLR4-induced STAT1 activation in THP-1 macrophages. Furthermore, the results demonstrated that both FP7 and FP12 significantly reduced TLR4-dependent STAT1 phosphorylation, but they did not affect exogenous IFN-β-induced STAT1 phosphorylation in THP-1 macrophages.

We obtained similar data using IP-10 production as a readout in THP-1 macrophages. The specific blockage of IFNAR prevented LPS from elevating IP-10 production (Figure 5d). FP7 and FP12 significantly reduced IP-10 production but they did not have any impact on exogenous IFN-β-induced IP-10 release from THP-1 macrophages (Figure 5c). These results suggest that FP7 and FP12 can block LPS-induced STAT1 activation via inhibition of IFN-β production in THP-1 macrophages.

## Discussion

Chronic inflammation has been documented as a critical event in a variety of inflammatory-related diseases. In this context, we have shown the essential role of TLR4 as a therapeutic target and suggest that the modulation of TLR4 signalling pathways will be beneficial for treatment.^17,18^ Pharmacological intervention of a variety of diseases using TLR4 antagonists has been a challenging approach for the last few decades. For example, Eritoran has previously been shown to prevent TLR4/TRIF-dependent signalling.^19^ However, these candidates failed in different stages of clinical trials.^20,21^ Therefore, the generation of novel TLR4 modulators, which are in different stages of pre-clinical or clinical validation, will be beneficial for the treatment in a broad range of inflammatory-related diseases including cancer.^22^

After a first generation of synthetic cationic amphiphiles (IAXO compounds) active in inhibiting TLR4 signal, our group developed a second generation of anionic, glycolipid-based TLR4 antagonists designated as FP molecules. As a result of the screening, we identified FP7 and FP12 as potent inhibitors of TLR4 which bind to MD-2 and displace LPS from TLR4 complex.^15^ Consequently, we investigated the molecular mechanisms by which these molecules affect TLR4 signalling in the context of inflammatory diseases.

Previously, we documented that the novel synthetic TLR4 antagonist FP7 inhibited TLR4 function and glycolytic re-programming of dendritic cells, and protected mice from death due to TLR4-dependent influenza infection.^23^ Interestingly, we showed that FP7 completely blocked the production of HMGB-1 (high mobility G box 1 protein, necrotic release factor)-induced TLR4-dependent pro-inflammatory cytokines from dendritic cells. These findings demonstrated that the negative regulation of TLR4 signalling by novel specific antagonists in response to DAMP-driven amplification of the immune response could be beneficial for virus-associated infectious diseases.

Moreover, we demonstrated that FP7 efficiently protected motor-neurons from LPS-induced lethality in spinal cord cultures, and inhibited IL-1β production from LPS-stimulated microglia in an amyotrophic lateral sclerosis mouse model.^24^ In another study, we showed that FP7 had the potential to inhibit haematopoietic and non-haematopoietic MyD88-dependent TLR4 signalling in response to distinct TLR4 ligands of sterile and non-sterile inflammation.^16^ Very recently, we reported the anti-inflammatory role of FP7 in inflammatory bowel disease (IBD).^25^ FP7 strongly reduced LPS-driven release of pro-inflammatory proteins in PBMCs isolated from IBD patients and *lamina propria* mononuclear cells isolated by biopsy of patients with Crohn’s disease and ulcerative colitis. Additionally, FP7 attenuated colonic inflammation in a mouse model of ulcerative colitis.^25^

In this study we further demonstrated the potential of the TLR4 antagonist FP7 and its novel derivative FP12 to block MyD88-independent TLR4 signalling in human macrophages. We showed the ability of FP7 and FP12 to negatively regulate the activity of second messengers (TBK1, IRF3 and STAT1) and IFN proteins production (IFN-β and IP-10) implicated in TRIF-dependent TLR4 signalling. Interestingly, we found differential properties of different structural variants of LPS to activate TRIF-dependent TLR4 pathways. While LPS forms purified from SM wild type or mutant strains and SM derived lipid A all activated MyD88-dependent TLR4 pathways, only S and Ra forms of LPS were linked with the activation of TRIF-dependent pathways in THP-1 macrophages. Additionally, we observed that the same properties of LPS forms are valid for activation of MyD88-dependent pathways based on p65 NF-kB phosphorylation (data is not shown). In support, meningococcal lipid A was shown as a weak agonist in stimulating TLR4/TRIF signalling in human macrophages,^26^ In contrast, monophosphoryl lipid A has been shown to activate human dendritic cells and peritoneal macrophages leading to type I IFN production.^26,27^

Importantly, we also showed that, irrespective of the method of administration (prior to or simultaneously with LPS stimulation), these small molecules inhibited the production of LPS/TLR4-driven IFN proteins (IFN-β and IP-10). Interestingly, we obtained similar results regarding MyD88-dependent TLR4 signalling in prior and simultaneous administration, where FP7 attenuated LPS stimulation in THP-1 macrophages (inhibiting the production of IL-1β and TNF-α. ^16^ While this negative effect on the production of IFN-β and IP-10 was also seen when FP7 was administered 30 min after LPS, FP12 reduced only IP-10 production, however IFN-β release did not appear to be affected *post factum*. This important pharmacological effect of FP7 and FP12 could be explained by competition between LPS and FP7/FP12 which bind to MD-2 and displace LPS.^13^ In this respect, we have already demonstrated the ability of FP7 to prevent LPS-driven TLR4 and CD14 internalisation, suggesting that FP7 can interact with both TLR4/MD-2 and CD14 and prevent activation of MyD88 and TRIF-dependent signalling in PBMC.^25^ Furthermore, the small differences in the potential of these small molecules to affect MyD88-dependent and MyD88-independent TLR4 signalling (administered 30 min after LPS) might be affected by internalisation of TLR4 in the cell cytoplasm. In this respect, it has been demonstrated that Rab10 is required for trafficking of TLR4 between the membrane and the Golgi apparatus, and TICAM-1/TICAM-2/TLR4 are essential for endosome localisation of TLR4-mediated IFN signalling.^28,29^ Furthermore, CD14 is required for LPS-induced TLR4 internalisation into endosomes and activation of TRIF pathways in macrophages.^30^ Additionally, CD14 deficiency completely blocked TBK1/IRF3 activation without affecting MyD88-dependent pathways. However, TLR4 endocytosis and TRIF pathways are activated by distinct ligands in the absence of CD14.^19^ Since Gram-negative pathogens produce both S- and R-forms of LPS, cells in the absence of membrane-bound or soluble CD14 will be able to recognise bacteria through R-form LPS. Evidence from the literature has demonstrated differential recognition of R- and S-forms of LPS by TLR4-MD-2. Freudenberg and colleagues demonstrated that R-form of LPS and free lipid A activated MyD88-dependent TLR4 signalling in the absence of CD14 and LPS-binding protein, which can lead to weaker activation of TRIF-dependent pathways.^31^ Huber and colleagues demonstrated that the Re-form can activate MyD88-dependent but not TRIF-dependent TLR4 pathways in THP-1 macrophages. Further studies to investigate the effect of FP7 and FP12 on downstream targets in TLR4 signalling, including TLR4 internalisation and degradation are needed.

In conclusion, the results from this study demonstrated the mechanism by which LPS/TLR4/TRIF signalling amplifies inflammatory response via type I IFN pathways in THP-1 macrophages. Furthermore, we showed that the synthetic TLR4 antagonists FP7 and FP12 were effective in blocking MyD88-independent TLR4 signalling, suggesting the potential of these small molecules for pharmacological intervention of inflammatory related diseases. Future work will be focused on pre-clinical validation of FP7 and FP12 for treatment of virus-related infectious disorders and sterile inflammation-driven diseases such as atherosclerosis and aneurysms.

## Supplemental Material

sj-pdf-1-ini-10.1177_17534259211005840 - Supplemental material for Synthetic glycolipid-based TLR4 antagonists negatively regulate TRIF-dependent TLR4 signalling in human macrophages Click here for additional data file. Supplemental material, sj-pdf-1-ini-10.1177_17534259211005840 for Synthetic glycolipid-based TLR4 antagonists negatively regulate TRIF-dependent TLR4 signalling in human macrophages by Charys Palmer, Fabio A Facchini, Richard PO Jones, Frank Neumann, Francesco Peri and Grisha Pirianov in Innate Immunity
