# Supplementary material for: Synthetic glycolipid-based TLR4 antagonists negatively regulate TRIF-dependent TLR4 signalling in human macrophages
Source: Innate Immun. 2021 Apr 16;27(3):275–84. doi: 10.1177/17534259211005840 (PMC8054148; doi:10.1177/17534259211005840)
Supplement: sj-pdf-1-ini-10.1177_17534259211005840 - Supplemental material for Synthetic glycolipid-based TLR4 antagonists negatively regulate TRIF-dependent TLR4 signalling in human macrophages [file sj-pdf-1-ini-10.1177_17534259211005840.pdf]

Supplement legends

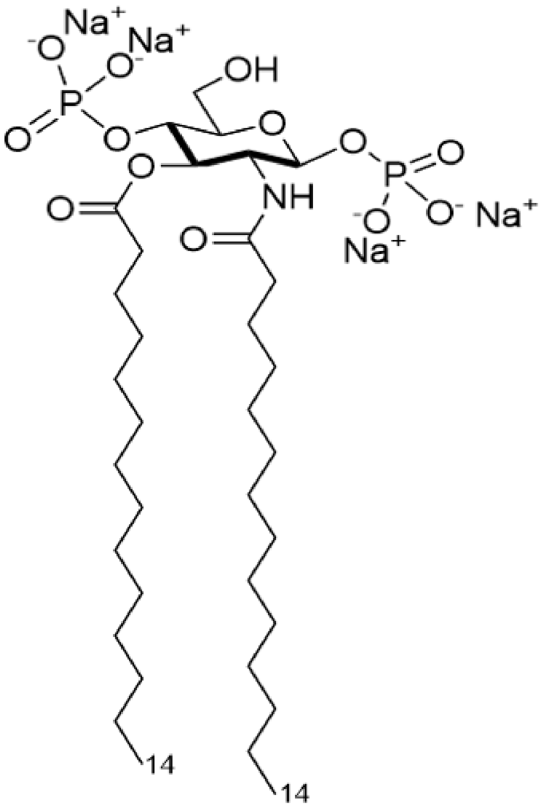

FP7

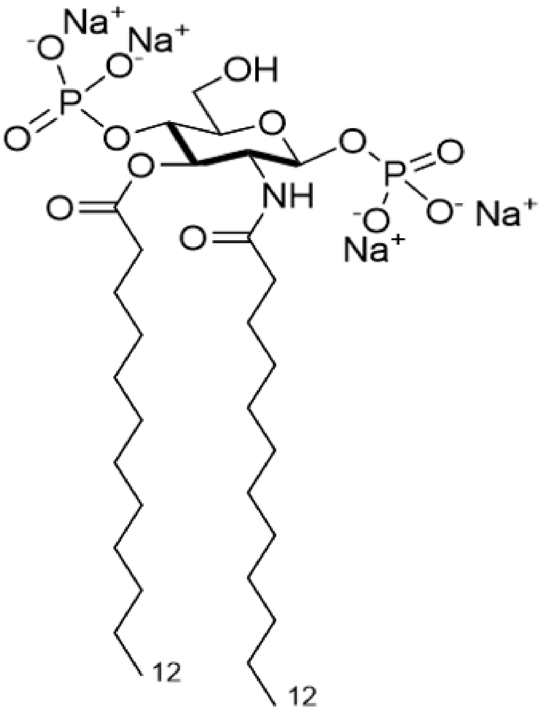

FP12

Figure 1S. Chemical structure of FP7 and FP12.

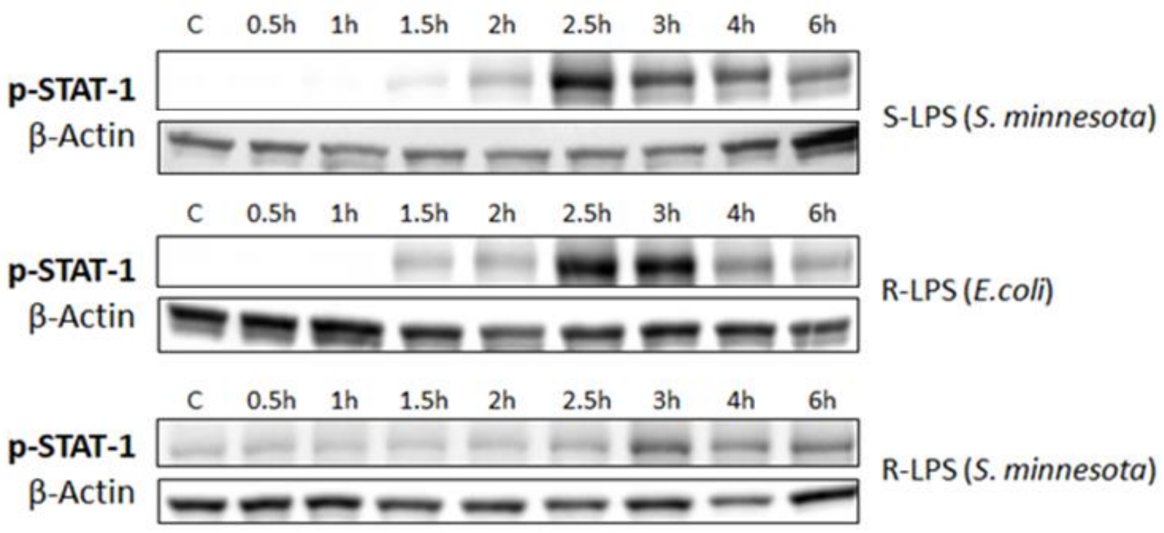

Figure 2S. Effect of different chemotypes of LPS as TLR4 ligands on STAT1 phosphorylation. THP-1 derived macrophages we exposed to LPS (SM, S- and Re-forms) and LPS (R-form *E. coli*). STAT1 phosphorylation was measured by Western blotting at (0-6) h. Actin was used as a loading control.

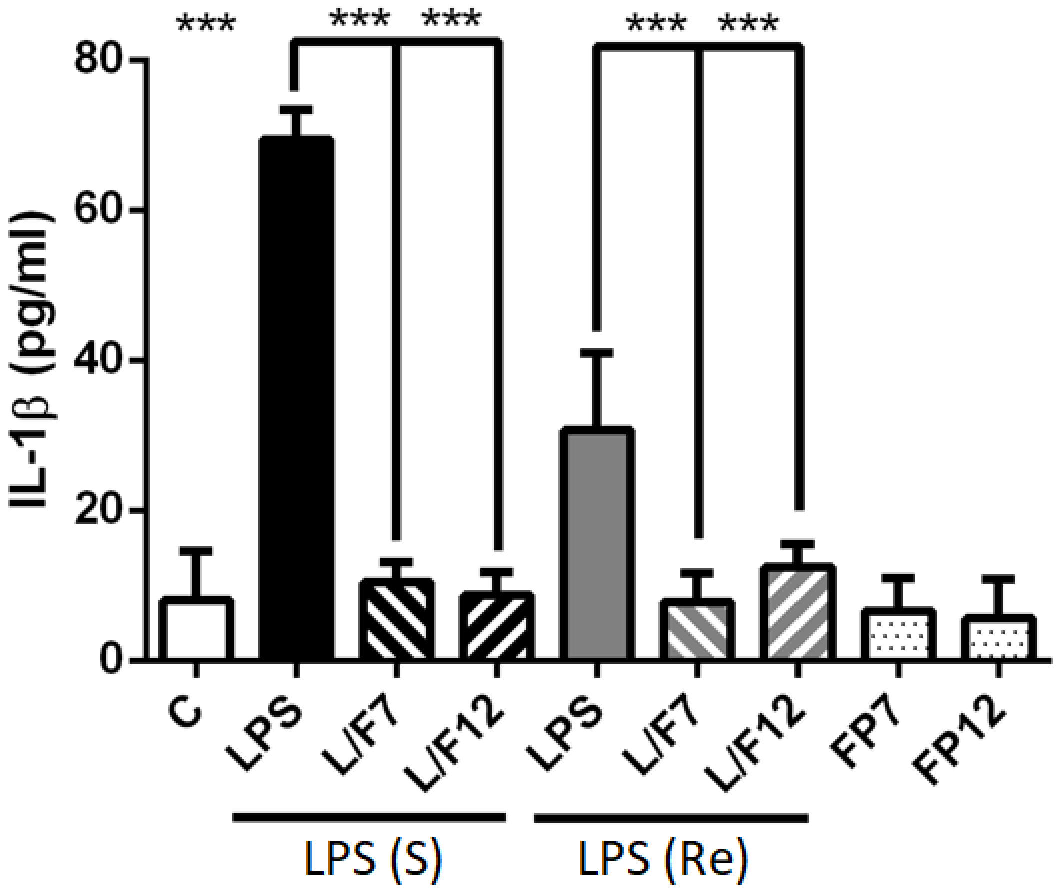

Figure 3S. FP7 and FP12 negatively regulate MyD88-dependent IL-1β production. THP-1-derived macrophages were pre-treated with FP7 (10 µM) or FP12 (10 µM) for 1 h before exposure to (S- and Re-forms) LPS (100 ng/ml). Cell medium was collected after 16 h and IL-1β production was measured by ELISA. Results are shown as mean ± SD of 3 independent experiments. Significant results are indicated as ****P* < 0.001 vs control.

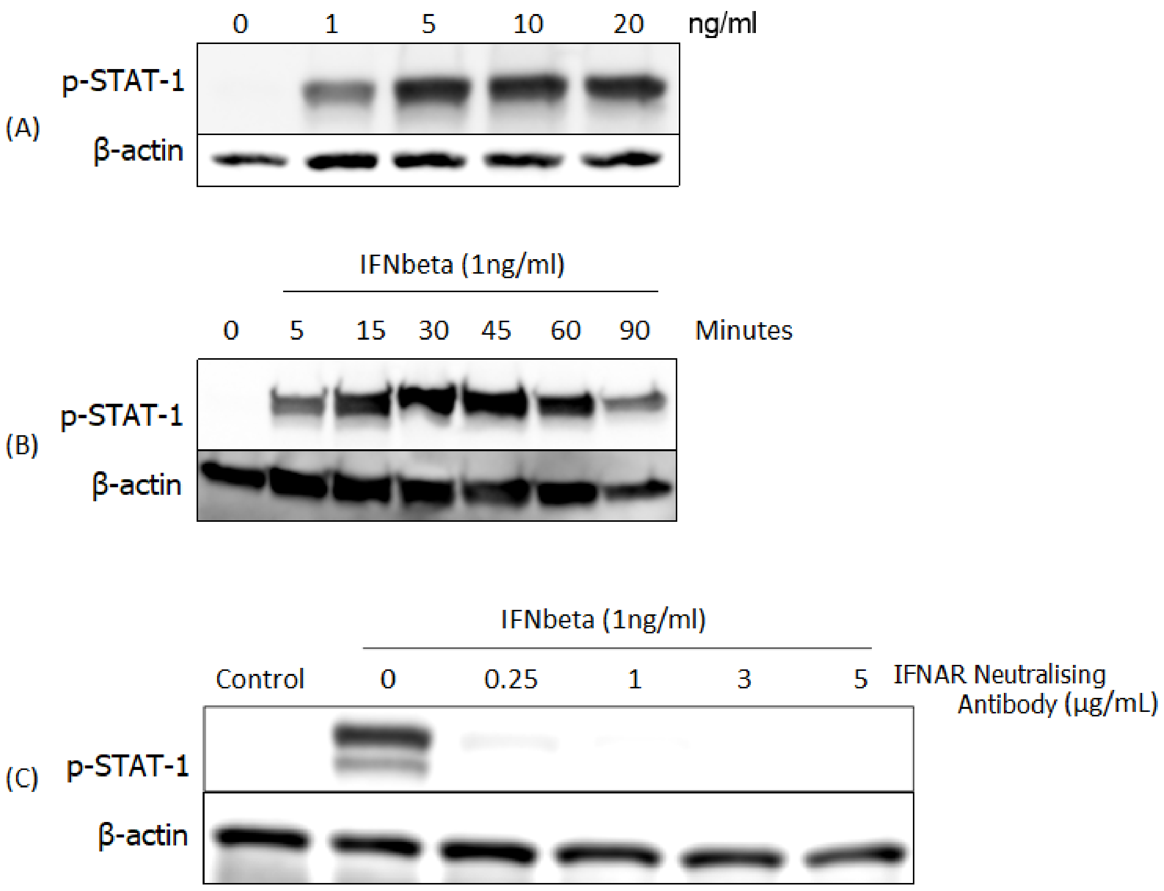

Figure 4S. Effect of IFN-β and IFNRA neutralising Ab on STAT1 phosphorylation in THP-1 macrophages. THP-1 derived macrophages were exposed to IFN-β (0-20 ng/ml) for 30 min (A). THP-1 derived macrophages were exposed to IFN-β (1 ng/ml) up to 90 min (B). THP-1 derived macrophages were pre-treated with IFNAR neutralising Ab (0-5 µg/ml) for 30 min and exposed to IFN-β (1 ng/ml) for additional 30 min (C). STAT1 phosphorylation was measured by Western blotting. Actin was used as a loading control.

Inflammatory Proteins	LPS	LPS/ FP7	LPS/ FP12	Inflammatory Proteins	LPS	LPS/ FP7	LPS/ FP12
1. EOTAXIN-1	5.95	0.97	1.10	21. IL-13	2.38	0.91	0.49
2. EOTAXIN-2	1.06	1.20	1.36	22. IL-15	0.71	0.58	0.83
3. GCSF	2.08	1.03	1.61	23. IL-16	1.53	1.09	1.34
4. GM-CSF	3.71	0.46	0.35	24. IL-17A	0.88	0.83	0.76
5. iCAM-1	3.21	1.59	2.09	25. IP-10	8.09	1.49	1.57
6. IFNγ	0.19	0.19	0.32	26. MCP-1	19.82	3.33	9.01
7. I-309	5.63	1.96	4.12	27. MCP-2	146.71	1.66	4.43
8. IL-1α	3.71	0.19	1.82	28. M-CSF	0.59	0.98	1.00
9. IL-1β	7.86	1.72	2.60	29. MIG	0.23	0.36	1.53
10. IL-2	0.45	1.09	1.18	30. MIP-1α	15.53	5.37	13.07
11. IL-3	1.03	1.05	1.07	31. MIP-1β	2.54	2.21	2.73
12. IL-4	4.69	0.84	0.95	32. MIP-1δ	1.39	0.68	0.44
13. IL-6	299.34	0.65	3.86	33. RANTES	0.98	0.83	0.85
14. IL-6R	1.60	2.03	2.30	34. TGFβ	0.83	0.86	0.89
15. IL-7	1.00	1.00	2.84	35. TNFα	15.88	0.93	1.31
16. IL-8	1.36	1.07	1.26	36. TNFβ	0.75	1.42	1.80
17. IL-10	7.00	1.42	3.34	37. TNF RI	1.31	1.27	1.19
18. IL-11	2.35	0.48	0.43	38. TNFII	1.39	1.10	1.15
19. IL-12p40	1.73	1.26	1.15	39. PGDFBB	0.25	2.31	0.93
20. IL-12p70	1.43	0.13	1.01	40. TIMP-2	1.02	1.09	0.98

(green) = > 2 fold increase vs control

(pink) = > 50% decrease vs LPS

Table 1S. Effect of FP7 and FP12 on TLR4-dependent pro-inflammatory proteins production in THP-1 derived macrophages. THP-1 macrophages were treated with FP7 or FP12 (10 µM) for 1 h prior to LPS (S-form) (100 ng/ml) exposure. Culture medium was collected after 18 h of incubation. A human inflammation array kit (Ray-Biotech, USA) was used to measure relative levels of cytokine expression between samples. Results values are expressed as fold-increase relative to control samples.
